# On the need for an international effort to capture, share and use crystallization screening data

**DOI:** 10.1107/S1744309112002618

**Published:** 2012-02-15

**Authors:** Janet Newman, Evan E. Bolton, Jochen Müller-Dieckmann, Vincent J. Fazio, D. Travis Gallagher, David Lovell, Joseph R. Luft, Thomas S. Peat, David Ratcliffe, Roger A. Sayle, Edward H. Snell, Kerry Taylor, Pascal Vallotton, Sameer Velanker, Frank von Delft

**Affiliations:** a Materials Science and Engineering, CSIRO, 343 Royal Parade, Parkville, VIC 3052, Australia; b NCBI, NLM, NIH, Department of Health and Human Services, 8600 Rockville Pike, Bethesda, MD 20894, USA; c EMBL Hamburg Outstation c/o DESY, Notkestrasse 85, D-22603 Hamburg, Germany; d National Institute for Standards and Technology, 9600 Gudelsky Drive, Rockville, MD 20850, USA; e CSIRO ICT Centre and CSIRO Mathematics, Informatics and Statistics, CS and IT Building 108, North Road, Australian National University, GPO Box 664, Canberra, ACT 2601, Australia; f Hauptman–Woodward Medical Research Institute, 700 Ellicott Street, Buffalo, NY 14203, USA; g SUNY Buffalo Department of Structural and Computational Biology, 700 Ellicott Street, Buffalo, NY 14203, USA; h NextMove Software, Innovation Center, Science Park, Milton Road, Cambridge CB4 0EY, England; i CSIRO Mathematics, Informatics and Statistics – North Ryde, Building E6B, Macquarie University Campus, North Ryde, NSW 1670, Australia; j EMBL Outstation Hinxton, European Bioinformatics Institute, Wellcome Trust Genome Campus, Hinxton, Cambridge CB10 1SD, England; k Structural Genomics Consortium, Oxford University, Old Road Campus Research Building, Old Road Campus, Roosevelt Drive, Oxford OX3 7DQ, England

**Keywords:** crystallization screening data, crystallization ontology

## Abstract

When crystallization screening is conducted many outcomes are observed but typically the only trial recorded in the literature is the condition that yielded the crystal(s) used for subsequent diffraction studies. The initial hit that was optimized and the results of all the other trials are lost. These missing results contain information that would be useful for an improved general understanding of crystallization. This paper provides a report of a crystallization data exchange (XDX) workshop organized by several international large-scale crystallization screening laboratories to discuss how this information may be captured and utilized. A group that administers a significant fraction of the world’s crystallization screening results was convened, together with chemical and structural data informaticians and computational scientists who specialize in creating and analysing large disparate data sets. The development of a crystallization ontology for the crystallization community was proposed. This paper (by the attendees of the workshop) provides the thoughts and rationale leading to this conclusion. This is brought to the attention of the wider audience of crystallographers so that they are aware of these early efforts and can contribute to the process going forward.

## Abstract 2

Development of an ontology for the description of crystallization experiments and results is proposed.

## 1. Introduction

‘Those who cannot remember the past are condemned to repeat it’ (Santayana, 1905 ▶).

Macromolecular crystallography has been extraordinarily productive as judged by the exponential growth of the database of structures, the Protein Data Bank (PDB; Berman *et al.*, 2007 ▶). That it has been judged to be a worthwhile pursuit for over half a century is shown by the continued support it receives from funding agencies around the world, by the almost universal demand for its results within the biochemical and molecular biology communities and by the prizes awarded to its practitioners, which include 11 Nobel Prizes.

The single most important requirement for structural experiments of this kind is the availability of appropriate crystals of the macromolecule of interest. This is as true now as when the first macromolecular structure was determined. Clearly, the impressive number of results captured by the PDB would not be possible if crystallization of macromolecules were impossible. But the quest for macromolecular crystals is currently a trial-and-error enterprise and it is perhaps surprising that so much structural biology has resulted from this approach to crystallization.

The concept of making crystallization more robust through the application of statistical tools was first published in 1979, in a seminal paper describing the use of factorial designs in a crystallization campaign (Carter & Carter, 1979 ▶). This paper has been widely cited, but the rigorous incomplete factorial methodology described in the paper has not been widely adopted by the crystallization community. The reasons can be found in the minutiae of the experiment described over three decades ago by Carter and Carter. The mathematics of incomplete design aren’t sensitive to the realities of a crystallization experiment: some crystallization factors are intrinsically coupled (pH and buffering species), and some combinations of independent factors are insoluble (Carter and Carter report that the combination of Mg^2+^ as a cation and PO_4_^3−^ as an anion stymied their analyses). Only six factor classes were used in their work (precipitant, anion, cation, divalent, temperature and pH) each with a very limited subset of factors. Furthermore, the methodology demanded that a ranked value is assigned to the result of each trial. Putting this all together, the Carter and Carter experiment was simply too difficult to be widely adopted. And yet the concept of rational exploration of crystallization space that is so well described in this paper continues to resonate within the community.

An even more widely cited paper (Jancarik & Kim, 1991 ▶) used the 1979 methodology as a springboard for creating a sparse matrix of crystallization conditions from a set of positive crystallization factors obtained from the literature. The sparse matrix was developed by trial and error, rather than through rigorous statistical balancing of the experiments, but overcame the problems of trying to fit pure statistics into the messy world of a working laboratory. The Jancarik and Kim paper revolutionized crystallization. The sparse matrix of crystallization conditions that they described was trivial to set up, and became even easier when the screen could be purchased as a set of pre-mixed solutions. The first commercial instance of the Jancarik and Kim screen was the ‘Crystal Screen’ from Hampton Research, available in 1991; this product is still available (product HR2-110). Since then, effectively all crystallization campaigns start by screening crystallization space using one or more of the hundreds of commercially available (Newman *et al.*, 2010 ▶) sparse matrix screens.

Many of the later sparse matrix screens have been developed by cherry-picking successful conditions. For example, the JCSG+ screen was derived from successful conditions obtained from a structural genomics project on *Thermotoga maritima* (Page *et al.*, 2003 ▶), and the Morpheus screen was derived from conditions associated with structures in the PDB (Gorrec, 2009 ▶). Are these second and third generation sparse matrix screens a sensible refinement of crystallization space, or are they artefacts of the community’s oversampling of a very limited number of points within a large crystallization space by the over-enthusiastic adoption of commercial screens? The structural biology community is certainly setting up many more crystallization experiments now than ever before. But does this mean the process of crystallization is now better or are we merely executing an ill-defined experiment more comprehensively? Certainly, the rate of producing structures has gone up (PDB, http://www.wwpdb.org), but is the improvement in efficiency a result of decreasing drop volumes? How much of the increase can be simply attributed to more people doing crystal structures? All these questions have been discussed previously (Rupp & Wang, 2004 ▶).

The use of automation has significantly increased the number of crystallization experiments and their associated data; every year there are literally millions of crystallization experiments being set up in laboratories worldwide. Despite this, it is still the case that the only data ever available externally from these efforts are the single crystallization conditions associated with successfully characterized structures, and often even these limited data are unavailable or ambiguous (Peat *et al.*, 2005 ▶; Tung & Gallagher, 2009 ▶). Other information is recorded, but we discard the vast bulk of this data including our experimental conditions and findings, so we cannot answer even simple questions of provenance or effort required. Of course, these data are not discarded wilfully, but only for lack of any effective use for them.

With these thoughts in mind, the authors (representatives from some of the larger public crystallization screening laboratories, experts in chemical notation and databases as well as computational scientists) came together in a small workshop in March 2011 led by the Commonwealth Scientific and Industrial Research Organization (CSIRO) in Canberra, Australia. The goal was to discuss ways that we might capture, share and learn by using all the information available from the vast number of crystallization experiments set up. In this paper we report on this workshop, in particular a discussion on how to capture crystallization experiments in a way that would help improve the success of not only our own crystallization efforts but also those of the community at large. We propose a crystallization ontology for the community and provide our thoughts and rationale leading to this conclusion. Our aim here is to bring this to the wider audience of crystallographers so that they are aware of our early efforts and can contribute to the process going forward.

## 2. Learning from ‘failure’

An experiment has only truly failed when it yields no information, rather than when its outcome fails to realise our hopes. This is not mere wordplay; this is the scientific method. However, in the high-throughput crystallization world, a rather narrow definition of ‘success’ has been adopted: initial screens are deemed a success if a crystal appeared; absence of crystals equates to ‘failure’. That we have become content with extracting a mere binary read-out from a set of hundreds of experiments, observed at multiple time-points as feature-rich images, should give us pause for thought.

Certainly experiments that do not produce crystals can be very informative. For instance, conditions that are not crystallization lead conditions (conditions immediately judged worthy of optimization) can guide us in determining where actual lead conditions are likely to lie. Non-crystalline outcomes provide valuable solubility data (Collins *et al.*, 2005 ▶), and the crystallization screen can be an effective method of understanding the phase behaviour of the sample (Snell *et al.*, 2008 ▶). Furthermore, information about the stability of the protein can be gleaned from these data as well. Simply knowing how much effort is normally required may guide decisions on when to move on to a different protein construct.

We can estimate how much information we lose with a too-narrow view of ‘failure’. The worldwide structural genomics efforts (where all outcomes, crystallization and non-crystallization, are tracked) show that out of ∼45K soluble, purified targets, ∼14K crystallized and ∼5K resulted in a crystal structure (Berman *et al.*, 2009 ▶). Another study, in one of our crystallization centres [the Hauptman-Woodward Medical Research Institute (HWI)], showed that a subset of 96 proteins screened against a set of 1536 chemical cocktails gave 277 crystal leads from ∼150K experiments. Although 36/96 of the proteins produced one or more crystals, this equates to only ∼0.2% of the experimental outcomes being crystals and ∼99.8% of the experiments producing some other outcome (Snell *et al.*, 2008 ▶).

The analysis above allows us to glimpse just how much data we are discarding, since data collated from the structural genomics efforts indicate the number of experiments associated with each structural success. If we start with ten purified, soluble protein constructs, then four are likely to crystallize, of which one is likely to produce a crystal structure. In the screening process described above, these ten proteins would be associated with 15 360 different crystallization screening experiments. Our 1536-well experiments suggest that an average of eight leads per sample are obtained when any individual lead condition is seen. For 15 360 different experiments and ten samples we average the results to ∼30 leads and 15 330 other outcomes, each of which adds more information on the protein’s behaviour. Extrapolating this using the ∼5000 crystal structures associated with structural genomics efforts, leads to an estimate of about 80 million other outcomes that are not captured. If we extrapolate it further to the whole PDB then the numbers become astronomical.

Most laboratories do not screen 1536 different chemical conditions in the initial search for lead conditions [for example the Collaborative Crystallization Centre (C3), the Hamburg High-Throughput Crystallization service (EMBL) and the Oxford Structural Genomics Consortium (SGC) use 384, 576 and 576 conditions, respectively^1^]. However, we have not counted any experiments associated with subsequent optimization of protocols, and we use data from the worldwide structural genomics efforts that may not represent the practices of an individual laboratory, where years of effort and experiments may be devoted to a particular project. Even if our numbers are only a crude estimate (say, accurate to within an order of magnitude), they demonstrate that we are missing data from tens to hundreds of millions of experiments. 

Clearly the combination of producing purified protein^2^ and crystallizing it is the major stumbling block in obtaining atomic resolution coordinates of proteins. We contend that access to neglected data is key to understanding the crystallization/protein production bottleneck, and furthermore that this requires a research effort beyond any individual laboratory. Tools need to be set in place so that data can be easily transferred, so we can avoid duplication of effort and achieve the required critical mass of investigation. Furthermore, by analysing data from a broad swath of laboratories we hope to capture many of the possible experimental techniques and results, thus making the output of such analyses widely applicable.

## 3. Attempts to data mine and improve crystallization

Consider the information provided by the analysis of limited crystallization data. In a binary study, looking at crystal or no crystal, Page *et al.* (2003 ▶) identified a minimal core screen. The Joint Center for Structural Genomics (JCSG) reported 392 out of 465 proteins (84%) required only 67 out of the 480 conditions sampled to yield a crystal hit. Remarkably, for samples reported from the University of Toronto (Kimber *et al.*, 2003 ▶) six biochemical conditions produced crystals for over half the proteins studied (180 out of 338). A simple analysis of crystal *versus* no crystal data identified a subset of conditions that, if used, had a high degree of success and could allow the exploration of other factors, *e.g.* sample concentration, additives *etc.* Interestingly a second paper from the same group (Collins *et al.*, 2005 ▶) explores the use of clear drops in determining buffers that may be particularly suited to crystallization. This example demonstrates that capturing data in a simple three-class system, *i.e.* crystal, clear, something else, provides information that can help the crystallization process. If we can expand this type of analysis to include a greater number of biochemically diverse proteins, and pay careful attention to bioinformatics associated with the sample, we can use this data to gain significant insight into the general process of crystallization.

Data on crystallization and subsequent X-ray diffraction from the North East Structural Genomics group were analysed (Price *et al.*, 2009 ▶). The analysis compared crystals that resulted in structures with bioinformatic and biophysical properties of the proteins. The data set consisted of 697 strongly expressed well behaved proteins with one construct for each protein target. These were screened to exclude samples that were aggregated, samples with predicted transmembrane α-helices or having greater than 20% low complexity sequences. Some 157 of these yielded crystal structures with an additional 39 yielding crystals that had insufficient diffraction for structural studies. The authors determined sequence specific features that correlated with crystallization propensity. Similarly, an analysis of data from the JCSG looked at protein production and crystallization; a set of 1503 proteins that were successfully crystallized and went on to reveal structural information were compared with 2456 that were not (Slabinski *et al.*, 2007 ▶). The authors also determined features that allowed an analysis of the potential for crystallization as it related to general biophysical properties of the sample. Each of these predictive mechanisms performs best when focused on the sample subset it has been trained upon. Expanding beyond the original data set to include more diverse samples requires analysis and testing of a population representing those samples. Similarly, expanding the capability will require expanded data. The authors note that for enhanced analysis ‘more effort on data standardization and exchange protocols is necessary’. An ontology approach achieves these two requirements.

A number of studies have discussed the most effective sampling strategy for crystallization. For example Segelke (2001 ▶) estimates that 288 trials are sufficient to find crystallization conditions with high probability and the studies reported above show that success with 6 or 67 conditions is still remarkably high. Almost immediately the reader should question the numbers we report: 384, 576 and 576 conditions with the extreme case being the HWI screening 1536 different chemical conditions (Luft *et al.*, 2003 ▶; Luft, Snell *et al.*, 2011 ▶) We contend that screening at this level is useful. Sampling more chemical space than that needed to find a single lead condition increases the probability of finding multiple conditions and provides information to guide subsequent optimization. In the HWI case, this was a deliberate design decision; the sampling of chemical space identifies not only crystallization conditions but also probes the protein’s solubility. Rupp (2003 ▶), considering protein crystallization as a sampling problem, noted that consistent data mining will be difficult because of inherent differences in the sampling of chemical space for screening and optimization, and the variety of crystallization methods employed. An ontology approach that takes these differences into account and allows a collective global analysis of the different crystallization practices in large centres and individual laboratories will be much more powerful than an individual analysis of experiments in a single centre.

## 4. Describing our attempts; measuring the outcomes

We identified two major, high-level challenges in achieving our ambition to capture crystallization data: how to describe our attempts to produce protein crystals; and how to measure the outcomes of these attempts.

A description of our trials in some unambiguous, reproducible and universally understood manner in principle requires nothing more than a set of standards and a way of ensuring compliance to them. Yet even this purely logistical, scientifically non-controversial task is very challenging, as there are currently no defined nomenclatures for describing a crystallization experiment, not for the chemicals used, nor for the physical parameters, never mind for the protein sample itself. Take, for example, the non-protein component of a ‘standard’ (vapour diffusion or microbatch) crystallization experiment; this has been called ‘the precipitant’, ‘the reservoir’, ‘the cocktail’, ‘the condition’, ‘the well solution’ or (for the hopeful) ‘the crystallant’ amongst others. It has been reported that in the free-form data field for crystallization in the PDB (REMARK 280), the chemical ‘ammonium sulfate’ is represented by approximately 100 different strings (Peat *et al.*, 2005 ▶).

The problem of capturing outcomes objectively would appear even more challenging still, as it requires scientific effort rather than merely establishing conventions. At least the push into high-throughput crystallization means that many recent experiments do have a measured outcome, in the form of one or more image(s) associated with the experiment. However, the image still has to be translated objectively into a form that can be used for quantitative analysis. This process will have to be automated to obtain not only complete but also consistent results: manual scoring of the same experiments is only about 70% consistent if using a seven-class system (Walker *et al.*, 2007 ▶).

The simplest outcome is the binary, crystal/no crystal classification, which can provide meaningful information. The other extreme is the classification of outcomes related to protein solubility and the phase diagram, *e.g.* crystal, clear, precipitate, phase separation, skin *etc.* (Luft, Wolfley *et al.*, 2011 ▶) which provides for significantly more information. This is a more detailed classification scheme, but requires correspondingly higher analysis times and the data will be less accurate than a simple binary classification. It becomes increasingly difficult to differentiate between the classifications of similar-looking outcomes when using a finer granularity in the classifications. Research in one of our centres (at HWI) has led to an automated classifier which is now comparable to humans at identifying single categories such as clear, precipitate, and also combinations of phase, skin, precipitate *etc.* but is not as precise or accurate when identifying crystals (Kotseruba *et al.*, 2012 ▶). Efforts to automate the classification of crystallization experimental outcomes have been ongoing for over a decade (Pan *et al.*, 2006 ▶; Cumbaa & Jurisica, 2005 ▶; Walker *et al.*, 2007 ▶). In designing our ontology we must keep in mind the reliability of the measurement and its associated data. We have to capture not only the outcome but how that outcome was determined. In this manner we can account for different visual mechanisms (multiple types of microscopes and magnifications) and classification schemes. These will be aided by using other parts of the light spectrum, *e.g.* ultraviolet, and even *in situ* X-ray analysis.

One of the results of the meeting was a commitment to develop a vocabulary to capture the complete crystallization data available to us. This vocabulary has several requirements. Overall it must be able to capture any crystallization experiment; not only those from the well defined protocols of large-scale crystallization centres, but also anything set up in less industrialized labs where scientists focus on individual projects. Regardless of where a crystallization experiment is performed, the information that needs to be captured is the same: we want to know about the sample, the experiment and the outcome, essentially the information captured in any good laboratory notebook. However, it is useful to consider this information in the context of what makes a difference to the experiment. Often seemingly small changes in a protocol can have a dramatic impact on the experiment’s outcome and reproducibility. Let us consider each of these three categories in detail.

The sample can be described by a name and the sequence of the protein,^3^ or proteins, that comprise it. Important protein properties may include sequence, molecular weight and isoelectric point (Slabinski *et al.*, 2007 ▶). The sample has other properties associated with it: even a minimal sample consisting of only one protein in water has an associated concentration, unit of concentration, a history (*e.g.* ‘snap frozen and thawed just prior to setup’). Preparation details of the sample may be also be important, *e.g.* ‘retention time on a column’, ‘purity’ and ‘polydispersity’.^3^

The experimental setup, even for something as common as a hanging-drop experiment (Benvenuti & Mangani, 2007 ▶), is also very hard to describe precisely. Assuming ‘hanging drop’, we need to know that is a type of ‘vapour diffusion’ and thus we should capture the chemicals used, drop volumes, reservoir volume, initial concentrations, predicted final concentrations, the time course, surface areas, geometry, material, incubation temperature, amongst other things. Indeed, even the time between drop mixing and sealing (in vapour diffusion), or the time course of temperature and dehydration can be critical.

Outcomes, the results of our experiments, are a morass into which we rarely delve with any enthusiasm: the sheer number of experiments which we don’t accurately describe, or describe at all, attests to this. To a large part this is a result of our fixation, almost a glori­fication, of crystals as the only useful result (Chayen & Saridakis, 2008 ▶). The non-crystal results can point toward an optimization direction, although one may have to work harder to determine what that direction is. There is a major difficulty in describing these non-crystalline outcomes. When does a precipitate become an amorphous or a crystalline precipitate? Is that drop clear, or is there evidence of a light precipitate? Even then we should note we are looking at results, and not reasons. Is that clear drop clear because it is under-saturated? Is it clear because it is metastable? Or does it appear clear because the perfect crystal contained within matches the refraction index of the surrounding liquid and we simply cannot see it? We should not only capture outcome, but also how that outcome was determined to add a level of confidence to the classification. Was the classification strictly an evaluation through a low-magnification binocular microscope, or were spectroscopic, UV fluorescence, light scattering, dyes, or other physico-chemical means employed for validation? One of the potential benefits of such rigour would be the development of metrics to allow us to abandon non-productive experiments early.

This emphasises that our vocabulary has to be comprehensive, it has to have multiple tiers to capture and integrate basic information recorded in one laboratory with more detailed information from another, and it has to be descriptive, precise and uniform.

A number of other disciplines have already faced these challenges leading to the development of computational analysis techniques built on ontologies (first seen as the New Latin *ontologia* ‘the study of that which is’). See for example Soldatova *et al.* (2006 ▶). An ontology can be described as a structured formalization of knowledge that reconciles different descriptions of similar things (Musen, 2007 ▶). Ontology development deals with questions concerning the entities of interest, and how they can be grouped, related to each other, and subdivided according to similarities and differences. By developing a common ontology, multiple different sets of data can be related to each other *via* a common descriptive language. Given the ontology as a basis, tools and methods of analysis developed for one set of data can be shared and directly applied to data from other groups.

The field of crystallography is not new to ontology developments. Under the auspices of the International Union of Crystallography a data exchange format was developed for small-molecule single-crystal diffraction experiments, the Crystallographic Information File (CIF) (Hall *et al.*, 1991 ▶). An extension to this for macromolecules (mmCIF) followed (Bourne *et al.*, 1997 ▶). This includes some terms for describing a successful crystal growth experiment but fewer for describing the unsuccessful majority of outcomes in a crystallization experiment. In developing a more detailed crystallization ontology, we will be building on the current mmCIF with the aim of developing a means to capture and be able to analyse all crystallization screening experiments. To do so we have to comprehensively define the ‘things’ that it needs to represent. This includes both physical objects, *e.g.* in the experiment example, ‘ammonium sulfate solution’, and the properties associated with the object, *e.g.* ‘3.14 *M* concentration’, ‘contains NH_4_^+^ ions’, ‘is volatile’, ‘has 2:1 stoichiometry of cations to anions’. The power in the ontology approach comes from the ability to use these descriptions and links between them (*e.g.* ‘all solutions containing the cation NH_4_^+^ are somewhat similar’) as the basis for both describing our experiments and understanding better the relationships between experimental conditions and outcomes.

## 5. Using an ontology

The goal of our ontology is to develop a common language for describing macromolecular crystallization experiments. We will improve communication and progress when we have a common nomenclature and universal descriptions that are shared by the community to capture the essence of the crystallization process. Once this is achieved, we have a common foundation to make all of our individual experiments accessible to others in the field. It is sobering that despite the structural victories enabled by the high-throughput technologies of the past decade, our means of sharing data is predominately through publications. Currently, even among seemingly similar crystallization platforms, we cannot move or readily assimilate experimental data. Although many of the high-throughput crystallization centres do analyse their own crystallization data, producing, amongst other things, screens which are combinations of experimentally derived hotspots of crystallization (Page *et al.*, 2003 ▶; Page & Stevens, 2004 ▶), these analyses are necessarily limited to the data from that centre. Once we step outside any individual centre, the best we can do in terms of data mining are rudimentary analyses of the collective, single crystallization conditions reported for structural determinations found in resources such as the PDB (Berman *et al.*, 2002 ▶, 2007 ▶), BMCD (Biological Macromolecular Crystallization Database; Tung & Gallagher, 2009 ▶), or MPCD (Marseille Protein Crystallization Database; Charles *et al.*, 2006 ▶), amongst others. We know of no other associated experimental details or results that are routinely captured and shared amongst crystallization laboratories. We need to develop a crystallization ontology to: enable a basic ability to share our data; permit cross-centre analyses; and explore the goal of learning from the non-crystalline outcomes that account for 99.8% of our experiments. Without an ontology, it is not clear if these goals can ever be realised. An immediate benefit of an ontology would be data standards which would help practitioners of crystallization to unambiguously describe their crystallization experiments. Thus, their results would be readily interpretable by other investigators, a point appreciated by anyone who has struggled to reproduce a crystal from published crystallization conditions.

We have commenced the task of building our ontology using the World Wide Web Consortium’s (W3C, http://www.w3.org/Consortium/) recommended Web Ontology Language, OWL. To define the scope of our ontology, we have employed the method proposed by Noy & McGuinness (2001 ▶) in which we design the ontology to address certain ‘competency questions’, some of which are shown below.

1. What was the outcome of this experiment (in qualitative terms)?
2. What were the methods used for this experiment?
3. What are the chemical and physical conditions of this experiment?
4. What is the chemical or physical relationship between the conditions of different experiments?
5. What observations relate to this experiment?
6. What sample was used for this experiment?
7. What was the intent of this batch of experiments?

Although there are a variety of tools available for developing ontologies, our initial modest efforts use the Web Ontology Language (OWL) for a number of reasons. It has the benefit of being well accepted; being highly structured, offering the advantages of both a formal schema and a controlled vocabulary; and importantly, being amenable to the representation of partial knowledge.

Currently, our ontology primarily comprises knowledge about chemical crystallants; what needs expansion is how to capture characteristics of proteins and constructs, experimental methods, conditions, and outcomes. Much of our chemical knowledge has been drawn from several pre-existing resources, including standard crystallization reference books, the IUPAC Gold Book, ChEBI, PubChem, and also incorporates dictionary terms published in the IUCr Macromolecular CIF dictionary (mmCIF), as well as drawing on our own unpublished knowledge of the field. However, we recognize that our initial attempt is incomplete, insufficiently documented, and is almost certainly at least partially incorrect.

One of the advantages of formalizing data in this manner is that we can begin to test machine-learning techniques to mine the large body of otherwise wasted experimental data. This will take time and a collective effort from the community. We invite our readers to contribute in the development of the ontology and invite them to collaborate; please visit http://xdx-ontology.org to take part. The eventual outcome will be to use the power of these massive quantities of collected experimental data to guide the most efficient crystallization of a single sample.

## 6. Summary

Crystallization of biological macromolecules is seen by most structural biologists as a necessary evil, a means to the end, which is knowledge about a biological system derived from a macromolecular structure. Although the focus of biologists may be on structural analysis to understand functional mechanisms, we argue that our current knowledge about getting to that point may be insufficient to meet the challenges of the future. We currently throw away much of the data that would otherwise enlighten us – not discarded lightly, but for lack of any efficient use for it. Without this option, to paraphrase our opening quote, ‘we are condemned to repeat the past rather than learning from it’. We believe that a concerted international effort needs to be made to establish a common means to capture, share and make use of this data. We hope that our initial efforts will help to start this process.

## Footnotes

1 The HWI, C3 and EMBL laboratories are services and will set up the number of experiments requested by the customer. Details are available at http://www.hwi.buffalo.edu/faculty_research/crystallization.html, http://www.csiro.au/c3 and http://www.embl-hamburg.de/facilities/htpx/, respectively.

2 Of course, great strides have been made in producing target macromolecules for positive crystallization results. The ability to clone specific genes and manipulate them (*via* PCR) in the 1970s and 1980s made much of modern crystallization possible. It may be that this area of endeavour might also benefit from an ontological analysis such as the one we propose.

3 The term ‘protein’ is used in this case to cover protein, nucleic acid and even the carbohydrate and small-molecule components of an experiment.
